# Supplementary material for: Co-Circulation of Two Independent Clades and Persistence of CHIKV-ECSA Genotype during Epidemic Waves in Rio de Janeiro, Southeast Brazil
Source: Pathogens. 2020 Nov 26;9(12):984. doi: 10.3390/pathogens9120984 (PMC7759993; doi:10.3390/pathogens9120984)
Supplement: Supplementary file 1 [file pathogens-09-00984-s001.zip › Supplementary_Files/Table S1.pdf]

Table S1. Sequences of the Chikungunya virus global dataset.

	Access Number (Genbank)	Country	Date (Year_Month_Day)	Genotype
1.	HM045786	Nigeria	1964_07_07	West African
2.	HM045798	Senegal	1966_11	West African
3.	HM045804	Senegal	NA	West African
4.	HM045816	Senegal	1966_11_23	West African
5.	HM045817	Senegal	2005_11	West African
6.	AB860301	Philippines	2013	Asian
7.	EU703759	Malaysia	2006	Asian
8.	EU703760	Malaysia	2006	Asian
9.	EU703761	Malaysia	2006	Asian
10.	EU703762	Malaysia	2006	Asian
11.	FJ807897	Indonesia	2007	Asian
12.	FN295483	Malaysia	2006_03	Asian
13.	FN295484	Malaysia	2006_03	Asian
14.	HE806461	New_Caledonia	2011_02_28	Asian
15.	HM045787	Thailand	1995	Asian
16.	HM045788	India	1973	Asian
17.	HM045789	Thailand	1988	Asian
18.	HM045790	Philippines	1985_07_17	Asian
19.	HM045791	Indonesia	1983	Asian
20.	HM045796	Thailand	1995	Asian
21.	HM045797	Indonesia	1985	Asian
22.	HM045800	Philippines	1985	Asian
23.	HM045803	India	1963_11_06	Asian
24.	HM045808	Thailand	1978	Asian
25.	HM045810	Thailand	1958	Asian
26.	HM045813	India	1963_11_06	Asian
27.	HM045814	Thailand	1975	Asian
28.	KC488650	China	2012	Asian
29.	KF318729	China	2012_07_06	Asian
30.	KF872195	Russia	2013_09_24	Asian
31.	KJ451622	Micronesia	2013_10	Asian
32.	KJ451623	Micronesia	2013_10	Asian
33.	KJ451624	British_Virgin_Islands	2014_01	Asian
34.	KM673291	Indonesia	2013_01	Asian
35.	KP164567	Brazil	2014_08_28	Asian
36.	KP164571	Brazil	2014_07_03	Asian
37.	KP164572	Brazil	2014_08_21	Asian
38.	KP851709	Mexico	2014_10_15	Asian
39.	KP851710	Mexico	2014_05_30	Asian
40.	KR046227	Trinidad_and_Tobago	2014_08_30	Asian
41.	KR046228	Trinidad_and_Tobago	2014_09_11	Asian
42.	KR046229	Trinidad_and_Tobago	2014_09_17	Asian
43.	KR046230	Trinidad_and_Tobago	2014_09_18	Asian
44.	KR046232	Trinidad_and_Tobago	2014_09_20	Asian
45.	KR046233	Trinidad_and_Tobago	2014_09_20	Asian
46.	KR046234	Trinidad_and_Tobago	2014_09_12	Asian
47.	KR264949	Puerto_Rico	2014_07_15	Asian

48.	KR264950	Puerto_Rico	2014_07_16	Asian
49.	KR264951	Puerto_Rico	2014_08_14	Asian
50.	KT192707	Nicaragua	2014_10_31	Asian
51.	KT308159	Philippines	2012	Asian
52.	KT308160	Philippines	2012	Asian
53.	KT308161	Philippines	2012	Asian
54.	KT308162	Philippines	2012	Asian
55.	KT308163	Philippines	2012	Asian
56.	KT581023	Brazil	2014	Asian
57.	KU355832	Brazil	2015	Asian
58.	KU365366	Mexico	2014_10_09	Asian
59.	KU365367	Mexico	2014_11_07	Asian
60.	KU365368	Mexico	2014_03_26	Asian
61.	KU365369	Trinidad_and_Tobago	2014	Asian
62.	KU365372	Colombia	2014_12_18	Asian
63.	KU365373	Colombia	2014_12	Asian
64.	KU365374	Venezuela	2014_12_17	Asian
65.	KX097982	Indonesia	2015	Asian
66.	KX097986	Indonesia	2015	Asian
67.	KX097988	Indonesia	2015	Asian
68.	KX168429	Malaysia	2009	Asian
69.	KX262987	Thailand	1996	Asian
70.	KX262988	Thailand	1988	Asian
71.	KX262991	Saint_Martin	2003	Asian
72.	KX262992	Guadeloupe	2014_01_05	Asian
73.	KX496989	Colombia	2016_02_09	Asian
74.	KX702401	Haiti	2014_06_02	Asian
75.	KX702402	Haiti	2014_06_09	Asian
76.	KY272961	Dominican_Republic	2014	Asian
77.	KY272963	Dominican_Republic	2014	Asian
78.	KY272964	Dominican_Republic	2014	Asian
79.	KY272965	Dominican_Republic	2014	Asian
80.	KY272966	Dominican_Republic	2014	Asian
81.	KY272967	Dominican_Republic	2014	Asian
82.	KY272968	Dominican_Republic	2014	Asian
83.	KY272969	Dominican_Republic	2014	Asian
84.	KY272970	Dominican_Republic	2014	Asian
85.	KY415985	Haiti	2014_08_13	Asian
86.	KY435454	Trinidad_and_Tobago	2011_11	Asian
87.	KY435455	Anguilla	2014_11_12	Asian
88.	KY435456	Suriname	2014_08_17	Asian
89.	KY435457	Montserrat	2014_10_30	Asian
90.	KY435458	Guyana	2014_11_03	Asian
91.	KY435459	Cayman_Islands	2014_09_17	Asian
92.	KY435460	Cayman_Islands	2014_07_06	Asian
93.	KY435461	Jamaica	2014_08_24	Asian
94.	KY435462	Jamaica	2014_08_25	Asian
95.	KY435463	Suriname	2014_08_02	Asian
96.	KY435464	Barbados	2014_08_15	Asian
97.	KY435465	Trinidad_and_Tobago	2014_08_17	Asian
98.	KY435466	Barbados	2014_08_06	Asian

99.	KY435467	Montserrat	2014_07_24	Asian
100.	KY435468	Jamaica	2014_08_06	Asian
101.	KY435469	Grenada	2014_07_30	Asian
102.	KY435470	Bahamas	2014_07_08	Asian
103.	KY435471	Turks_and_Caicos_Islands	2014_06_11	Asian
104.	KY435472	Grenada	2014_06_16	Asian
105.	KY435473	Saint_Lucia	2014_05_22	Asian
106.	KY435474	Saint_Lucia	2014_05_19	Asian
107.	KY435475	Saint_Vincent_and_the_Grenadines	2014_05_22	Asian
108.	KY435476	Turks_and_Caicos_Islands	2014_06_05	Asian
109.	KY435477	Guyana	2014_05_31	Asian
110.	KY435478	Guyana	2014_05_17	Asian
111.	KY435479	Antigua_and_Barbuda	2014_04_28	Asian
112.	KY435480	Haiti	2014_04_27	Asian
113.	KY435481	Saint_Lucia	2014_04_22	Asian
114.	KY435482	Saint_Kitts_and_Nevis	2014_03_11	Asian
115.	KY435483	Anguilla	2014_02_12	Asian
116.	KY435484	Dominica	2014_01_30	Asian
117.	KY435485	Dominica	2014_01_28	Asian
118.	KY435486	British_Virgin_Islands	2014_01_23	Asian
119.	KY575565	USA	2014	Asian
120.	KY575566	USA	2014	Asian
121.	KY575569	USA	2014	Asian
122.	KY575572	USA	2014	Asian
123.	KY575573	USA	2014	Asian
124.	KY680348	USA	2014_09_05	Asian
125.	KY680349	USA	2014_07_02	Asian
126.	KY680350	USA	2014_12_10	Asian
127.	KY680351	USA	2014_06_24	Asian
128.	KY680352	USA	2014_05_15	Asian
129.	KY680353	USA	2014_08_25	Asian
130.	KY680354	USA	2014_05_08	Asian
131.	KY680355	USA	2014_07_22	Asian
132.	KY680356	USA	2014_07_21	Asian
133.	KY680357	USA	2014_08_09	Asian
134.	KY680358	USA	2014_08_08	Asian
135.	KY680359	USA	2014_07_04	Asian
136.	KY680360	USA	2014_08_05	Asian
137.	KY680361	USA	2014_05_26	Asian
138.	KY680362	USA	2014_07_26	Asian
139.	KY680363	USA	2014_06_15	Asian
140.	KY680365	USA	2014_09_18	Asian
141.	KY680366	USA	2015_07_24	Asian
142.	KY680367	USA	2014_06_09	Asian
143.	KY680368	USA	2014_12_01	Asian
144.	KY680369	USA	2014_05_24	Asian
145.	KY680370	USA	2014_10_03	Asian
146.	KY680371	USA	2014_08_20	Asian
147.	KY680372	USA	2014_06_04	Asian
148.	KY680373	USA	2014_10_22	Asian
149.	KY680374	USA	2014_12_06	Asian

150.	KY680375	USA	2014_06_04	Asian
151.	KY680376	USA	2014_10_08	Asian
152.	KY680377	USA	2014_08_16	Asian
153.	KY680378	USA	2014_10_19	Asian
154.	KY680379	USA	2014_09_16	Asian
155.	KY680380	USA	2014_10_13	Asian
156.	KY680381	USA	2014_10_21	Asian
157.	KY680382	USA	2014_06_04	Asian
158.	KY680383	USA	2014_08_20	Asian
159.	KY680384	USA	2014_06_25	Asian
160.	KY680385	USA	2014_11_25	Asian
161.	KY680386	USA	2014_09_24	Asian
162.	KY680387	USA	2014_10_14	Asian
163.	KY680388	USA	2014_10_02	Asian
164.	KY680389	USA	2015_07_14	Asian
165.	KY680390	USA	2014_05_07	Asian
166.	KY680391	USA	2014_10_24	Asian
167.	KY680392	USA	2014_10_19	Asian
168.	KY680393	USA	2014_09_17	Asian
169.	KY680394	USA	2014_10_05	Asian
170.	KY680395	USA	2014_07_21	Asian
171.	KY680396	USA	2014_09_02	Asian
172.	KY680397	USA	2014_05_23	Asian
173.	KY680398	USA	2014_08_05	Asian
174.	KY680399	USA	2014_09_05	Asian
175.	KY680400	USA	2014_07_15	Asian
176.	KY680401	USA	2014_09_22	Asian
177.	KY680402	USA	2014_07_27	Asian
178.	KY680403	USA	2014_06_14	Asian
179.	KY680404	USA	2014_09_17	Asian
180.	KY680405	USA	2014_06_18	Asian
181.	KY680406	USA	2014_08_08	Asian
182.	KY680407	USA	2014_11_20	Asian
183.	KY680408	USA	2014_06_26	Asian
184.	KY680409	USA	2014_10_21	Asian
185.	KY680410	USA	2014_08_22	Asian
186.	KY680411	USA	2014_09_04	Asian
187.	KY680412	USA	2014_08_28	Asian
188.	KY680413	USA	2014_09_17	Asian
189.	KY680414	USA	2014_09_13	Asian
190.	KY703888	Nicaragua	2015_08_15	Asian
191.	KY703889	Nicaragua	2015_08_05	Asian
192.	KY703890	Nicaragua	2015_09_23	Asian
193.	KY703891	Nicaragua	2015_09_17	Asian
194.	KY703892	Nicaragua	2015_11_26	Asian
195.	KY703893	Nicaragua	2015_09_02	Asian
196.	KY703894	Nicaragua	2015_10_09	Asian
197.	KY703895	Nicaragua	2015_11_26	Asian
198.	KY703896	Nicaragua	2014_12_18	Asian
199.	KY703897	Nicaragua	2015_01_16	Asian
200.	KY703898	Nicaragua	2015_08_25	Asian

201.	KY703899	Nicaragua	2015_10_20	Asian
202.	KY703900	Nicaragua	2015_12_14	Asian
203.	KY703901	Nicaragua	2015_01_26	Asian
204.	KY703902	Nicaragua	2015_09_05	Asian
205.	KY703903	Nicaragua	2015_09_05	Asian
206.	KY703904	Nicaragua	2014_10_04	Asian
207.	KY703905	Nicaragua	2015_09_17	Asian
208.	KY703906	Nicaragua	2015_09_08	Asian
209.	KY703907	Nicaragua	2015_07_15	Asian
210.	KY703908	Nicaragua	2014_12_02	Asian
211.	KY703909	Nicaragua	2015_01_28	Asian
212.	KY703910	Nicaragua	2015_10_18	Asian
213.	KY703911	Nicaragua	2015_12_17	Asian
214.	KY703912	Nicaragua	2015_12_14	Asian
215.	KY703913	Nicaragua	2015_08_13	Asian
216.	KY703914	Nicaragua	2015_08_15	Asian
217.	KY703915	Nicaragua	2015_08_03	Asian
218.	KY703916	Nicaragua	2015_08_04	Asian
219.	KY703917	Nicaragua	2016_01_08	Asian
220.	KY703918	Nicaragua	2015_01_26	Asian
221.	KY703919	Nicaragua	2015_01_29	Asian
222.	KY703920	Nicaragua	2015_11_16	Asian
223.	KY703921	Nicaragua	2015_11_04	Asian
224.	KY703922	Nicaragua	2015_09_06	Asian
225.	KY703923	Nicaragua	2015_09_19	Asian
226.	KY703924	Nicaragua	2015_08_13	Asian
227.	KY703925	Nicaragua	2015_09_06	Asian
228.	KY703926	Nicaragua	2015_08_20	Asian
229.	KY703927	Nicaragua	2015_01_15	Asian
230.	KY703928	Nicaragua	2015_01_30	Asian
231.	KY703929	Nicaragua	2015_12_02	Asian
232.	KY703930	Nicaragua	2015_11_24	Asian
233.	KY703931	Nicaragua	2015_09_19	Asian
234.	KY703932	Nicaragua	2015_08_19	Asian
235.	KY703933	Nicaragua	2015_10_09	Asian
236.	KY703934	Nicaragua	2015_10_27	Asian
237.	KY703935	Nicaragua	2015_12_02	Asian
238.	KY703936	Nicaragua	2015_10_07	Asian
239.	KY703937	Nicaragua	2015_10_15	Asian
240.	KY703938	Nicaragua	2015_02_18	Asian
241.	KY703939	Nicaragua	2015_12_01	Asian
242.	KY703940	Nicaragua	2014_10_28	Asian
243.	KY703941	Nicaragua	2015_10_28	Asian
244.	KY703942	Nicaragua	2015_08_04	Asian
245.	KY703943	Nicaragua	2015_10_10	Asian
246.	KY703944	Nicaragua	2015_10_03	Asian
247.	KY703945	Nicaragua	2015_10_02	Asian
248.	KY703946	Nicaragua	2015_10_12	Asian
249.	KY703947	Nicaragua	2014_11_19	Asian
250.	KY703948	Nicaragua	2015_08_18	Asian
251.	KY703949	Nicaragua	2015_01_15	Asian

252.	KY703950	Nicaragua	2014_11_25	Asian
253.	KY703951	Nicaragua	2015_08_05	Asian
254.	KY703953	Nicaragua	2015_10_06	Asian
255.	KY703954	Nicaragua	2014_11_05	Asian
256.	KY703955	Nicaragua	2015_08_17	Asian
257.	KY703956	Nicaragua	2014_10_28	Asian
258.	KY703957	Nicaragua	2015_09_05	Asian
259.	KY703958	Nicaragua	2015_11_25	Asian
260.	KY703959	Nicaragua	2014_12_03	Asian
261.	KY703960	Nicaragua	2015_08_05	Asian
262.	KY703961	Nicaragua	2015_08_06	Asian
263.	KY703962	Nicaragua	2015_10_01	Asian
264.	KY703963	Nicaragua	2015_09_07	Asian
265.	KY703964	Nicaragua	2015_10_16	Asian
266.	KY703965	Nicaragua	2015_09_03	Asian
267.	KY703966	Nicaragua	2015_12_10	Asian
268.	KY703967	Nicaragua	2015_08_12	Asian
269.	KY703968	Nicaragua	2015_01_23	Asian
270.	KY703969	Nicaragua	2014_11_14	Asian
271.	KY703970	Nicaragua	2015_11_28	Asian
272.	KY703971	Nicaragua	2015_11_28	Asian
273.	KY703972	Nicaragua	2014_12_09	Asian
274.	KY703973	Nicaragua	2015_11_06	Asian
275.	KY703974	Nicaragua	2015_09_03	Asian
276.	KY703975	Nicaragua	2015_09_22	Asian
277.	KY703976	Nicaragua	2015_09_05	Asian
278.	KY703977	Nicaragua	2015_08_03	Asian
279.	KY703978	Nicaragua	2015_12_02	Asian
280.	KY703979	Nicaragua	2015_09_22	Asian
281.	KY703980	Nicaragua	2015_09_06	Asian
282.	KY703981	Nicaragua	2014_11_21	Asian
283.	KY703982	Nicaragua	2015_11_30	Asian
284.	KY703983	Nicaragua	2014_12_11	Asian
285.	KY703984	Nicaragua	2014_11_27	Asian
286.	KY703985	Nicaragua	2015_08_13	Asian
287.	KY703986	Nicaragua	2015_09_16	Asian
288.	KY703987	Nicaragua	2015_11_04	Asian
289.	KY703988	Nicaragua	2014_11_27	Asian
290.	KY703989	Nicaragua	2014_12_03	Asian
291.	KY703990	Nicaragua	2015_11_21	Asian
292.	KY703991	Nicaragua	2015_10_09	Asian
293.	KY703992	Nicaragua	2015_08_05	Asian
294.	KY703993	Nicaragua	2014_11_07	Asian
295.	KY703994	Nicaragua	2015_11_18	Asian
296.	KY703995	Nicaragua	2015_08_13	Asian
297.	KY703996	Nicaragua	2015_12_26	Asian
298.	KY703997	Nicaragua	2015_12_19	Asian
299.	KY703998	Nicaragua	2015_08_06	Asian
300.	KY703999	Nicaragua	2015_01_19	Asian
301.	KY704000	Nicaragua	2015_08_05	Asian
302.	KY704001	Nicaragua	2015_01_21	Asian

303.	KY704002	Nicaragua	2015_01_15	Asian
304.	KY883764	Singapore	2013_01	Asian
305.	LC259082	Thailand	1958	Asian
306.	LC259083	Indonesia	2009_09_11	Asian
307.	LC259084	Philippine	2012_09_25	Asian
308.	LC259085	Indonesia	2012_10_15	Asian
309.	LC259086	Indonesia	2013_07_01	Asian
310.	LC259087	Indonesia	2013_07_06	Asian
311.	LC259088	Tonga	2014_03_25	Asian
312.	LC259089	Dominica	2014_06_23	Asian
313.	LC259090	Colombia	2015_01_06	Asian
314.	LC259091	Indonesia	2015_05_07	Asian
315.	LC259092	Cuba	2016_02_28	Asian
316.	LC500215	Aruba	2015_01	Asian
317.	LC500216	Aruba	2015_01	Asian
318.	LC500217	Aruba	2015_01	Asian
319.	LC500218	Aruba	2015_01	Asian
320.	LC500219	Aruba	2015_01	Asian
321.	LC500220	Aruba	2015_01	Asian
322.	LC500221	Aruba	2015_02	Asian
323.	LC500222	Aruba	2015_03	Asian
324.	LN898093	Martinique	2013_12	Asian
325.	LN898094	Martinique	2014_01	Asian
326.	LN898095	Martinique	2014_01	Asian
327.	LN898096	Martinique	2014_01	Asian
328.	LN898097	Martinique	2014_01	Asian
329.	LN898098	Martinique	2014_01	Asian
330.	LN898100	Martinique	2014_01	Asian
331.	LN898101	Martinique	2014_01	Asian
332.	LN898103	Martinique	2014_01	Asian
333.	LN898104	Martinique	2014_01	Asian
334.	LN898105	Martinique	2014_01	Asian
335.	LN898107	Martinique	2014_01	Asian
336.	LN898108	Martinique	2014_01	Asian
337.	LN898110	Martinique	2014_01	Asian
338.	LN898111	Martinique	2014_01	Asian
339.	MF001505	USA	2015	Asian
340.	MF001506	USA	2015	Asian
341.	MF001507	USA	2015	Asian
342.	MF001508	USA	2015	Asian
343.	MF001509	USA	2015	Asian
344.	MF001510	USA	2015	Asian
345.	MF001511	USA	2015	Asian
346.	MF001512	USA	2015	Asian
347.	MF001513	USA	2015	Asian
348.	MF001514	USA	2015	Asian
349.	MF001515	USA	2015	Asian
350.	MF001516	USA	2015	Asian
351.	MF001517	USA	2015	Asian
352.	MF001518	USA	2015	Asian
353.	MF001519	USA	2015	Asian

354.	MF773559	Samoa	2014	Asian
355.	MF773560	NA	2014	Asian
356.	MF773561	Indonesia	2011	Asian
357.	MF773562	Kiribati	2015	Asian
358.	MF773563	Philippines	2014	Asian
359.	MF773564	Philippines	2016	Asian
360.	MF773565	Timor_Leste	2010	Asian
361.	MG208125	Saint_Martin	2013	Asian
362.	MG664851	China	2012	Asian
363.	MG921596	Mexico	2015	Asian
364.	MG967666	Haiti	2014_06	Asian
365.	MH329293	Colombia	2014_11	Asian
366.	MH329294	Colombia	2014_10_15	Asian
367.	MH329295	Colombia	2014_11	Asian
368.	MH329296	Colombia	2014_09_13	Asian
369.	MH329297	Colombia	2014_11	Asian
370.	MH329298	Colombia	2014_11	Asian
371.	MH329299	Colombia	2014_11	Asian
372.	MH329300	Colombia	2014_11_03	Asian
373.	MH329301	Colombia	2014_10_01	Asian
374.	MH329303	Colombia	2014_10_01	Asian
375.	MH359139	Colombia	2014_10_15	Asian
376.	MH359140	Colombia	2014_11_04	Asian
377.	MH359142	Colombia	2014_10_15	Asian
378.	MH670649	China	2009_11_19	Asian
379.	MK028840	Thailand	1962	Asian
380.	MK134712	Brazil	2014_12_03	Asian
381.	MK134713	Brazil	2014_11_11	Asian
382.	HM045792	South_Africa	1956_04	ECSA
383.	HM045795	South_Africa	1976	ECSA
384.	HM045809	Democratic_Republic_of_the_Congo	1960	ECSA
385.	HM045811	Tanzania	1953_02_22	ECSA
386.	HM045812	Uganda	1982	ECSA
387.	HM045822	Central_African_Republic	1978_10	ECSA
388.	KJ679577	India	2011_09_12	ECSA
389.	KP003812	Gabon_2007	2007	ECSA
390.	KP003813	Republic_of_the_Congo	2011	ECSA
391.	KP164568	Brazil-BA	2014	ECSA
392.	KP164568	Brazil	2014_08_26	ECSA
393.	KP164569	Brazil	2014_08_28	ECSA
394.	KP164570	Brazil	2014_09_03	ECSA
395.	KU940225	Brazil	2015_07_15	ECSA
396.	KU940226	Brazil	2015_08_01	ECSA
397.	KX228391	Brazil	2016_03_03	ECSA
398.	KX262996	Cameroon	2006	ECSA
399.	KY038947	Central_African_Republic	1983_12	ECSA
400.	KY124328	Brazil	2016_03_16	ECSA
401.	KY124329	Brazil	2016_03_16	ECSA
402.	KY575574	USA	1995	ECSA
403.	KY704933	Brazil	2016_03_30	ECSA
404.	KY704934	Brazil	2016_03_30	ECSA

405.	KY704935	Brazil	2016_04_01	ECSA
406.	KY704936	Brazil	2016_04_01	ECSA
407.	KY704937	Brazil	2016_04_15	ECSA
408.	KY704938	Brazil	2016_04_19	ECSA
409.	KY704939	Brazil	2016_04_17	ECSA
410.	KY704940	Brazil	2016_04_14	ECSA
411.	KY704941	Brazil	2016_04_14	ECSA
412.	KY704942	Brazil	2016_04_16	ECSA
413.	KY704943	Brazil	2016_04_19	ECSA
414.	KY704944	Brazil	2016_04_19	ECSA
415.	KY704945	Brazil	2016_04_14	ECSA
416.	KY704946	Brazil	2016_04_17	ECSA
417.	KY704947	Brazil	2016_04_15	ECSA
418.	KY704948	Brazil	2016_04_19	ECSA
419.	KY704949	Brazil	2016_04_07	ECSA
420.	KY704950	Brazil	2016_04_07	ECSA
421.	KY704951	Brazil	2016_04_13	ECSA
422.	KY704952	Brazil	2016_04_07	ECSA
423.	KY704953	Brazil	2016_04_09	ECSA
424.	KY704954	Brazil	2016_06_20	ECSA
425.	KY704955	Brazil	2016_06_17	ECSA
426.	LC259094	Angola	2016_05_11	ECSA
427.	MH823663	Brazil	2017_01_05	ECSA
428.	MH823664	Brazil	2017_03_16	ECSA
429.	MH823665	Brazil	2017_03_16	ECSA
430.	MH823666	Brazil	2017_03_01	ECSA
431.	MH823667	Brazil	2017_03_01	ECSA
432.	MH823668	Brazil	2017_01_05	ECSA
433.	MK121891	Brazil	2015_07_15	ECSA
434.	MK121892	Brazil	2015_07_15	ECSA
435.	MK121893	Brazil	2015_07_15	ECSA
436.	MK121894	Brazil	2016_01_31	ECSA
437.	MK121895	Brazil	2017_03_20	ECSA
438.	MK121896	Brazil	2017_03_03	ECSA
439.	MK121897	Brazil	2017_02_20	ECSA
440.	MK121898	Brazil	2017_02_22	ECSA
441.	MK121899	Brazil	2017_03_17	ECSA
442.	MK121900	Brazil	2017_03_17	ECSA
443.	MK121901	Brazil	2017_03_17	ECSA
444.	MK121902	Brazil	2017_03_17	ECSA
445.	MK121903	Brazil	2017_03_15	ECSA
446.	MK121904	Brazil	2017_03_02	ECSA
447.	MK121905	Brazil	2017_03_02	ECSA
448.	MK121906	Brazil	2017_02_27	ECSA
449.	MK121907	Brazil	2017_02_27	ECSA
450.	MK121908	Brazil	2017_03_05	ECSA
451.	MK244632	Brazil	2016_04_05	ECSA
452.	MK244633	Brazil	2016_05_06	ECSA
453.	MK244634	Brazil	2016_04_27	ECSA
454.	MK244635	Brazil	2016_02_19	ECSA
455.	MK244636	Brazil	2016_05_02	ECSA

456.	MK244637	Brazil	2016_05_10	ECSA
457.	MK244638	Brazil	2016_04_05	ECSA
458.	MK244639	Brazil	2016_04_19	ECSA
459.	MK244640	Brazil	2017_03_07	ECSA
460.	MK244641	Brazil	2017_03_09	ECSA
461.	MK244642	Brazil	2018_02_18	ECSA
462.	MK244643	Brazil	2018_03_28	ECSA
463.	MK244644	Brazil	2018_03_26	ECSA
464.	MK244645	Brazil	2018_03_28	ECSA
465.	MK244646	Brazil	2018_03_28	ECSA
466.	MK244647	Brazil	2018_03_27	ECSA
467.	MK244648	Brazil	2018_04_04	ECSA
468.	MK244649	Brazil	2018_04_03	ECSA
469.	MK244650	Brazil	2018_04_03	ECSA
470.	MK244651	Brazil	2018_04_05	ECSA
471.	MK244652	Brazil	2018_04_05	ECSA
472.	MK244653	Brazil	2018_04_06	ECSA
473.	MK244654	Brazil	2018_04_06	ECSA
474.	MK244655	Brazil	2018_04_06	ECSA
475.	MK244656	Brazil	2018_04_06	ECSA
476.	MK690206	Republic_of_the_Congo	2019	ECSA
477.	MK752950	Brazil	2017_08_23	ECSA
478.	MK752951	Brazil	2017_07_27	ECSA
479.	MK752952	Brazil	2017_08_30	ECSA
480.	MK752953	Brazil	2017_08_16	ECSA
481.	MK752954	Brazil	2017_06_22	ECSA
482.	MK752955	Brazil	2017_06_05	ECSA
483.	MK752956	Brazil	2017_08_30	ECSA
484.	MK752958	Brazil	2017_08_23	ECSA
485.	MK935343	Republic_of_the_Congo	2019_03_20	ECSA
486.	MK935344	Republic_of_the_Congo	2019_03_05	ECSA
487.	AB455493	NA	2006_12	IOL
488.	AB455494	NA	2006_12	IOL
489.	DQ443544	Reunion	NA	IOL
490.	EF210157	India	2006	IOL
491.	EU372006	India	2007_06_11	IOL
492.	EU564334	Mauritius	2006_02_14	IOL
493.	EU564335	India	2006_10	IOL
494.	FJ000062	India	2006_09	IOL
495.	FJ000063	India	2006_10	IOL
496.	FJ000064	India	2006_09	IOL
497.	FJ000065	India	2006_09	IOL
498.	FJ000066	India	2006_09	IOL
499.	FJ000067	India	2006_08	IOL
500.	FJ000068	India	2006_08	IOL
501.	FJ000069	India	2007_06	IOL
502.	FJ445426	Sri_Lanka	2008_04	IOL
503.	FJ445427	Sri_Lanka	2007_07	IOL
504.	FJ445428	Sri_Lanka	2007_05	IOL
505.	FJ445430	Singapore	2008_07	IOL
506.	FJ445431	Singapore	2008_07	IOL

507.	FJ445432	Singapore	2008_07	IOL
508.	FJ445433	Singapore	2008_08	IOL
509.	FJ445443	Singapore	2008_08	IOL
510.	FJ445445	Singapore	2008_08	IOL
511.	FJ445463	Singapore	2008_07	IOL
512.	FJ445484	Singapore	2008_05	IOL
513.	FJ445502	Singapore	2008_08	IOL
514.	FJ445510	Singapore	2008_01	IOL
515.	FJ445511	Singapore	2008_01	IOL
516.	FJ513628	Sri_Lanka	2008_03	IOL
517.	FJ513629	Sri_Lanka	2008_03	IOL
518.	FJ513632	Sri_Lanka	2008_03	IOL
519.	FJ513635	Sri_Lanka	2008_03	IOL
520.	FJ513637	Sri_Lanka	2008_03	IOL
521.	FJ513645	Sri_Lanka	2008_04	IOL
522.	FJ513654	Sri_Lanka	2008_04	IOL
523.	FJ513657	Sri_Lanka	2008_04	IOL
524.	FJ513673	Sri_Lanka	2008_04	IOL
525.	FJ513675	Sri_Lanka	2008_04	IOL
526.	FJ513679	Sri_Lanka	2008_04	IOL
527.	FJ807896	Singapore	2006	IOL
528.	FJ807898	Bangladesh	2008	IOL
529.	FJ807899	Malaysia	2008	IOL
530.	FJ959103	Mauritius	2006	IOL
531.	FN295485	Malaysia	2008	IOL
532.	FN295487	Malaysia	2008	IOL
533.	FR687340	Malaysia	2008_08_21	IOL
534.	FR687341	Malaysia	2008_11_05	IOL
535.	FR687342	Malaysia	2008_11_10	IOL
536.	FR687343	Malaysia	2008_12_12	IOL
537.	FR687344	Malaysia	2009_01_12	IOL
538.	FR687345	Malaysia	2009_02_05	IOL
539.	FR687346	Malaysia	2009_02_16	IOL
540.	FR687347	Malaysia	2009_02_12	IOL
541.	FR687348	Malaysia	2009_04_03	IOL
542.	FR717336	France	2005_12_26	IOL
543.	FR717337	France	2005_12_26	IOL
544.	GQ428210	India	2006_10_07	IOL
545.	GQ428211	India	2006_10_07	IOL
546.	GQ428212	India	2007_07_12	IOL
547.	GQ428213	India	2007_07_13	IOL
548.	GQ428214	India	2008_06_29	IOL
549.	GQ428215	India	2008_05_29	IOL
550.	GQ905863	Thailand	2009_05_25	IOL
551.	GU013528	Sri_Lanka	2008_03	IOL
552.	GU013529	Sri_Lanka	2008_03	IOL
553.	GU013530	Sri_Lanka	2008_04	IOL
554.	GU189061	Sri_Lanka	2006	IOL
555.	GU199350	China	2008	IOL
556.	GU199351	China	2008	IOL
557.	GU199352	China	2008	IOL

558.	GU199353	China	2008	IOL
559.	GU301779	Thailand	2009_09_04	IOL
560.	GU301780	Thailand	2008_10_21	IOL
561.	GU301781	Thailand	2009_07_27	IOL
562.	HM045794	USA	2006	IOL
563.	HM045799	Sri_Lanka	2007	IOL
564.	HM045801	Sri_Lanka	2007	IOL
565.	HQ846356	China	2010_10	IOL
566.	HQ846357	China	2010_10	IOL
567.	HQ846358	China	2010_10	IOL
568.	HQ846359	China	2010_10	IOL
569.	JF274082	India	2006_09_27	IOL
570.	JN558834	India	2009	IOL
571.	JN558835	India	2008	IOL
572.	JN558836	India	2009	IOL
573.	JQ065885	China	2010_10	IOL
574.	JQ065886	China	2010_10	IOL
575.	JQ065887	China	2010_10	IOL
576.	JQ065888	China	2010_10	IOL
577.	JQ065889	China	2010_10	IOL
578.	JQ065890	China	2010_10	IOL
579.	JQ065891	China	2010_10	IOL
580.	JQ065892	China	2010_10	IOL
581.	JQ861253	Cambodia	2011_08_16	IOL
582.	JQ861254	Cambodia	2011_08_16	IOL
583.	JQ861255	Cambodia	2011_08_16	IOL
584.	JQ861256	Cambodia	2011_08_16	IOL
585.	JQ861257	Cambodia	2011_08_16	IOL
586.	JQ861258	Cambodia	2011_08_16	IOL
587.	JQ861259	Cambodia	2011_05_26	IOL
588.	JQ861260	Cambodia	2011_05_28	IOL
589.	JX088705	China_2010	2010	IOL
590.	KC862329	Indonesia	2010	IOL
591.	KF151174	Myanmar	2009_07_13	IOL
592.	KF151175	Myanmar	2009_12_11	IOL
593.	KF283986	Comoros	2005	IOL
594.	KF283987	Comoros	2005	IOL
595.	KF590564	Myanmar	2010	IOL
596.	KF590565	Myanmar	2010	IOL
597.	KF590566	Myanmar	2010	IOL
598.	KF590567	Myanmar	2010	IOL
599.	KJ579184	Thailand	2013_10_14	IOL
600.	KJ579185	Thailand	2013_10_14	IOL
601.	KJ579186	Thailand	2013_10_14	IOL
602.	KJ579187	Thailand	2013_10_14	IOL
603.	KJ679578	India	2011_12_21	IOL
604.	KJ941050	USA	2006	IOL
605.	KP003807	France	2006	IOL
606.	KP003808	Madagascar	2006	IOL
607.	KP003809	Mayotte	2006	IOL
608.	KP003810	Italy	2007	IOL

609.	KP003811	Italy	2007	IOL
610.	KP702297	Comoros	2005	IOL
611.	KT324224	Malaysia	2008_12	IOL
612.	KT324225	Malaysia	2009_01	IOL
613.	KT324226	Malaysia	2009_01	IOL
614.	KT324227	Malaysia	2009_03	IOL
615.	KT324228	Malaysia	2009_02	IOL
616.	KT449801	Reunion	NA	IOL
617.	KU365370	Bangladesh	2011_11	IOL
618.	KU365371	Bangladesh	2011_11	IOL
619.	KX009167	Thailand	2013	IOL
620.	KX009168	Thailand	2013	IOL
621.	KX009169	Thailand	2013	IOL
622.	KX009170	Thailand	2013	IOL
623.	KX009171	Thailand	2013	IOL
624.	KX262989	Italy	2007	IOL
625.	KX262993	Italy	2007	IOL
626.	KX262997	Malaysia	2009	IOL
627.	KX619422	India	2014_07_08	IOL
628.	KX619423	India	2014_07_23	IOL
629.	KX619424	India	2015_09_22	IOL
630.	KX619425	India	2015_09_11	IOL
631.	KX619426	India	2015_12_03	IOL
632.	KX881784	India	NA	IOL
633.	KY057363	India	2016_08_28	IOL
634.	KY575567	USA	2006	IOL
635.	KY575568	USA	2006	IOL
636.	KY575570	USA	2008	IOL
637.	KY575571	USA	2006	IOL
638.	KY751908	Australia	2006	IOL
639.	LC259093	Malaysia	2009_01_06	IOL
640.	LC331252	Japan	2016	IOL
641.	MF076568	Laos	2013_03_28	IOL
642.	MF076569	Laos	2013_03_28	IOL
643.	MF076570	Laos	2013_03_28	IOL
644.	MF076571	Laos	2013_03_28	IOL
645.	MF076572	Laos	2013_03_12	IOL
646.	MF076573	Laos	2012_08_07	IOL
647.	MF076574	Laos	2012_08_16	IOL
648.	MF076575	Laos	2012_08_16	IOL
649.	MF076576	Laos	2012_08_31	IOL
650.	MF499120	Hong_Kong	2016_09_15	IOL
651.	MF503628	Hong_Kong	2016_08_26	IOL
652.	MF740874	Pakistan	2017	IOL
653.	MF773566	Bangladesh	2017	IOL
654.	MF773567	Borneo	2011	IOL
655.	MF773568	Malaysia	2008	IOL
656.	MF773569	Papua_New_Guinea	2013	IOL
657.	MF774613	Pakistan	2016	IOL
658.	MF774614	Pakistan	2016	IOL
659.	MF774615	Pakistan	2016	IOL

660.	MF774616	Pakistan	2016	IOL
661.	MF774617	Pakistan	2016	IOL
662.	MF774618	Pakistan	2016	IOL
663.	MF774619	Pakistan	2016	IOL
664.	MG049915	Italy	2017	IOL
665.	MG137428	India	2016_06_29	IOL
666.	MG664850	China	2010	IOL
667.	MG912993	China	2017_08_30	IOL
668.	MG925665	China	2017_12_15	IOL
669.	MH124570	India	2010	IOL
670.	MH124571	India	2010	IOL
671.	MH124572	India	2010	IOL
672.	MH124573	India	2010	IOL
673.	MH124574	India	2010	IOL
674.	MH124575	India	2010	IOL
675.	MH124576	India	2010	IOL
676.	MH124577	India	2010	IOL
677.	MH124578	India	2010	IOL
678.	MH124579	India	2010	IOL
679.	MH124580	India	2016	IOL
680.	MH124581	India	2016	IOL
681.	MH124582	India	2016	IOL
682.	MH124583	India	2016	IOL
683.	MH400249	China	2017_08_23	IOL
684.	MH423797	Kenya	2016_05_07	IOL
685.	MH423798	Kenya	2016_05_08	IOL
686.	MH423799	Kenya	2016_05_31	IOL
687.	MH423800	Kenya	2016_05_31	IOL
688.	MH423801	Kenya	2016_05_31	IOL
689.	MH423802	Kenya	2016_05_31	IOL
690.	MH423803	Kenya	2016_05_31	IOL
691.	MH423804	Kenya	2016_06_01	IOL
692.	MH423805	Kenya	2016_06_01	IOL
693.	MH423806	Kenya	2016_06_01	IOL
694.	MH423810	Kenya	2016_05_31	IOL
695.	MH507158	Italy	2017	IOL
696.	MK028838	Sri_Lanka	2006	IOL
697.	MK086029	Sri_Lanka	2006_11_14	IOL
698.	MK120195	Italy	2017_09_27	IOL
699.	MK120197	Italy	2017_08_02	IOL
700.	MK120198	Italy	2017_08_02	IOL
701.	MK120199	Italy	2017_08_02	IOL
702.	MK120200	Italy	2017_08_09	IOL
703.	MK120201	Italy	2007_08_20	IOL
704.	MK120202	Italy	2007_08_25	IOL
705.	MK163628	Sudan	2018_10	IOL
706.	MK370030	India	2015	IOL
707.	MK370031	India	2015	IOL
708.	MK370032	India	2016	IOL
709.	MK370033	India	2016	IOL
710.	MK468608	Bangladesh	2017_06_17	IOL

711.	MK468609	Bangladesh	2017_06_19	IOL
712.	MK468610	Bangladesh	2017_11_22	IOL
713.	MK468611	Bangladesh	2017_05_31	IOL
714.	MK468612	Bangladesh	2017_06_17	IOL
715.	MK468613	Bangladesh	2017_06_24	IOL
716.	MK468614	Bangladesh	2017_07_10	IOL
717.	MK468615	Bangladesh	2017_07_10	IOL
718.	MK468616	Bangladesh	2017_06_19	IOL
719.	MK468617	Bangladesh	2017_06_21	IOL
720.	MK468618	Bangladesh	2017_06_29	IOL
721.	MK468619	Bangladesh	2017_07_08	IOL
722.	MK468620	Bangladesh	2017_07_11	IOL
723.	MK468621	Bangladesh	2017_07_18	IOL
724.	MK468622	Bangladesh	2017_08_31	IOL
725.	MK468623	Bangladesh	2017_05_31	IOL
726.	MK468624	Bangladesh	2017_06_14	IOL
727.	MK468625	Bangladesh	2017_05_31	IOL
728.	MK468626	Bangladesh	2017_06_17	IOL
729.	MK468627	Bangladesh	2017_09_18	IOL
730.	MK468801	Thailand	2018_06_27	IOL
731.	MK473621	India	2016_09_02	IOL
732.	MK473622	India	2016_09_13	IOL
733.	MK473623	India	2016_09_20	IOL
734.	MK473624	India	2016_10_23	IOL
735.	MK473625	India	2016_08_26	IOL
736.	MK473626	India	2016_10_06	IOL
737.	MK473627	India	2016_10_23	IOL
738.	MK473628	India	2016_10_13	IOL
739.	MK473629	India	2016_10_18	IOL
740.	MK473630	India	2016	IOL
741.	MK473631	India	2016_09_11	IOL
742.	MK473632	India	2016_09_17	IOL
743.	MK473633	India	2016_10_19	IOL
744.	MK473634	India	2016	IOL
745.	MK473635	India	2016_09_02	IOL
746.	MK473636	India	2016_09_20	IOL
747.	MK473637	India	2016_09_20	IOL
748.	MK473638	India	2016_09_20	IOL
749.	MK473639	India	2016_10_23	IOL
750.	MK473640	India	2016_10_18	IOL
751.	MK518340	India	2016_09_28	IOL
752.	MK551552	India	2016_09_30	IOL
753.	MK551553	India	2016_10_03	IOL
754.	MK848202	Thailand	2018_11_21	IOL
755.	MN075149	Finland	2019_02	IOL
756.	MN075150	Finland	2019_02	IOL
757.	MN402883	China	2019_05_07	IOL
758.	MN402884	China	2019_06_28	IOL
759.	MN402885	China	2019_05_08	IOL
760.	MN402886	China	2019_07_02	IOL
761.	MN402887	Myanmar	2019_08_01	IOL

762.	MN402888	Myanmar	2019_08_01	IOL
763.	MN402889	China	2019_07_25	IOL
764.	MN402890	China	2019_07_26	IOL
765.	MN402891	China	2019_07_30	IOL
766.	MN402892	China	2019_08_02	IOL
767.	MN630017	Australia	2019	IOL
