# Supplementary material for: Co-Circulation of Two Independent Clades and Persistence of CHIKV-ECSA Genotype during Epidemic Waves in Rio de Janeiro, Southeast Brazil
Source: Pathogens. 2020 Nov 26;9(12):984. doi: 10.3390/pathogens9120984 (PMC7759993; doi:10.3390/pathogens9120984)
Supplement: Supplementary file 1 [file pathogens-09-00984-s001.zip › Supplementary_Files/Figure S1.pdf]

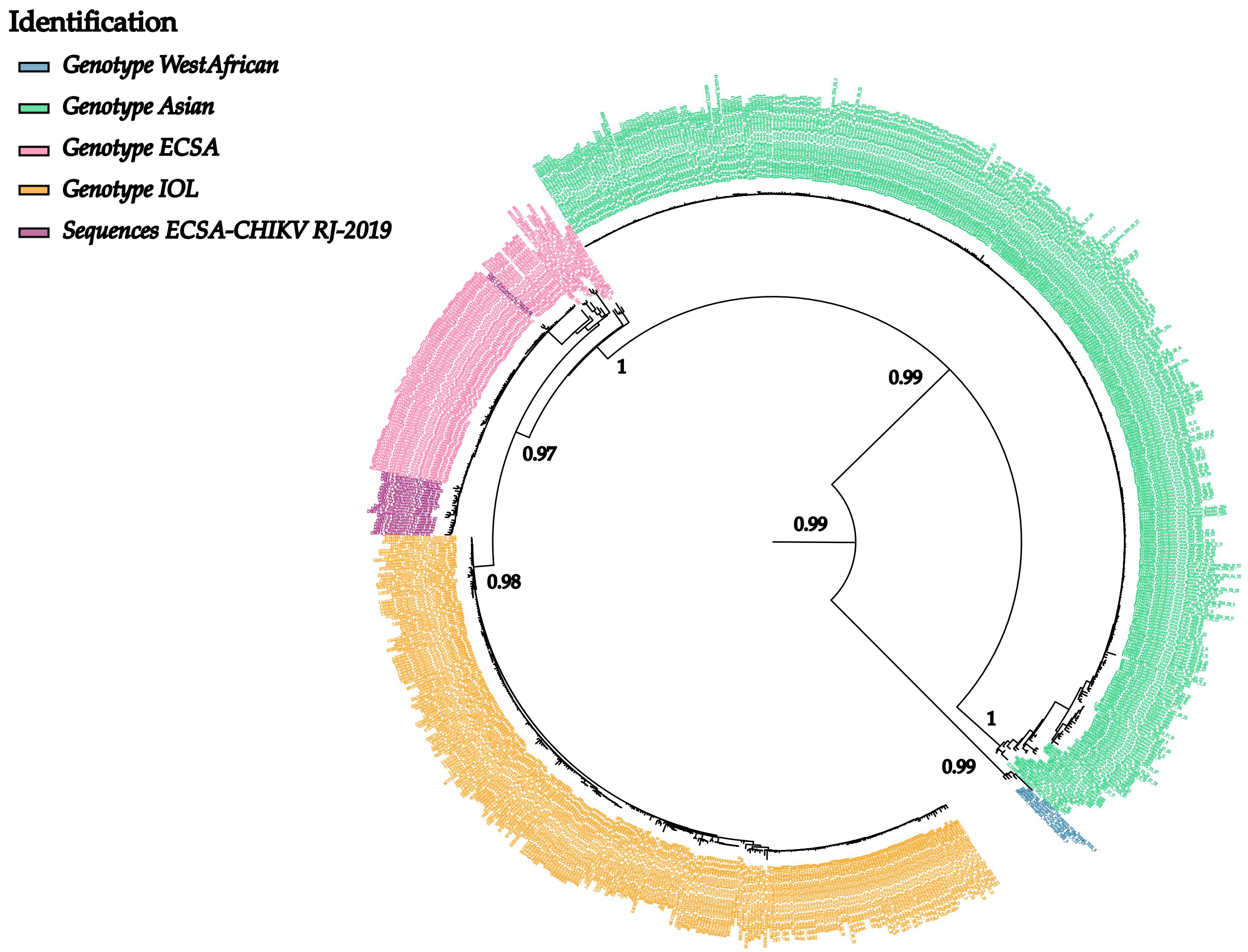

Identification
Genotype WestAfrican
Genotype Asian
Genotype ECSA
Genotype IOL
Sequences ECSA-CHIKV RJ-2019
1
0.99
0.97
0.99
0.98
1
0.99
